# Volume assessment accuracy in computed tomography: a phantom study

**DOI:** 10.1120/jacmp.v11i2.3037

**Published:** 2010-04-16

**Authors:** Nicolas D. Prionas, Shonket Ray, John M. Boone

**Affiliations:** ^1^ Department of Radiology University of California Davis Medical Center Sacramento CA USA

**Keywords:** computed tomography, volumetric, accuracy, phantom

## Abstract

There is a broad push in the cancer imaging community to eventually replace linear tumor measurements with three‐dimensional evaluation of tumor volume. To evaluate the potential accuracy of volume measurement in tumors by CT, a gelatin phantom consisting of 55 polymethylmethacrylate (PMMA) spheres spanning diameters from 1.6 mm to 25.4 mm was fabricated and scanned using thin slice (0.625 mm) CT (GE LightSpeed 16). Nine different reconstruction combinations of field of view dimension (FOV=20,30,40 cm) and CT kernel (standard, lung, bone) were analyzed. Contiguous thin‐slice images were averaged to produce CT images with greater thicknesses (1.25, 2.50, 5.0 mm). Simple grayscale thresholding techniques were used to segment the PMMA spheres from the gelatin background, where a total of 1800 spherical volumes were evaluated across the permutations studied. The geometric simplicity of the phantom established upper limits on measurement accuracy. In general, smaller slice thickness and larger sphere diameters produced more accurate volume assessment than larger slice thickness and smaller sphere diameter. The measured volumes were smaller than the actual volumes by a common factor depending on slice thickness; overall, 0.625 mm slices produced on average 18%, 1.25 mm slices produced 22%, 2.5 mm CT slices produced 29%, and 5.0 mm slices produced 39% underestimates of volume (mm^3^). Field of view did not have a significant effect on volume accuracy. Reconstruction algorithm significantly affected volume accuracy (p<0.0001), with the lung kernel having the smallest error, followed by the bone and standard kernels. The results of this investigation provide guidance for CT protocol development and may guide the development of more advanced techniques to promote quantitatively accurate CT volumetric analysis of tumors.

PACS number: 87.57.Q‐

## I. INTRODUCTION

Tumor volume change in response to therapy is believed to be a prognostic indicator of therapeutic success.^(^ ^1^ ^–^ ^3^ ^)^ Currently, the standard for monitoring tumor size is outlined in the joint guidelines from the European Organization for Research and Treatment of Cancer (EORTC) and the National Cancer Institutes of the United States and Canada, entitled Response Evaluation Criteria in Solid Tumors (RECIST).^(4)^ These guidelines expand on the previous recommendations by the World Health Organization (WHO), published in 1979. RECIST, published in 2000, describes a method of using medical imaging, particularly the reproducible images obtained with computed tomography (CT) and magnetic resonance imaging (MRI), to measure the longest diameter of a given target lesion, or the sum of the longest diameters for a set of target lesions, before and after therapy. This method is time‐efficient and versatile but, fundamentally, it is a one‐dimensional measurement, poorly representing the true morphology and size of a lesion.^(5)^ ^,^ ^(6)^ Furthermore, it does not take into consideration the effects of various scan and reconstruction parameters on image acquisition and analysis.^(7)^ ^,^ ^(8)^ While appropriate at the time of its introduction, the simplicity of RECIST now underutilizes the sophisticated advances in modern CT imaging.

With the advent of helical CT, improvements in detector performance, and the use of narrow slice thicknesses below 1 mm, the ability to assess tumor volume using three‐dimensional metrics has become much more feasible. While hand‐outlining regions of interest remains the gold‐standard for segmentation, improvements in computer‐aided segmentation and other image analysis packages look to provide alternatives to the inter‐observer variability reported in previous studies.^(^ ^9^ ^–^ ^11^ ^)^ However, before volume‐based metrics can supplement RECIST, these methods must be shown to be accurate and precise. Similarly, the effects of various scan and reconstruction parameters on accuracy and precision must be further characterized. Indeed, RECIST version 1.1 was released in 2009, and it stressed the importance of studying volumetric anatomical assessment in greater detail before anatomic unidimensional assessment of tumor burden can be abandoned.^(12)^

The purpose of this study was to assess the effects of slice thickness, reconstruction field of view (FOV), and reconstruction kernel on the accuracy of CT‐based volume estimation over a range of object sizes typical of solid tumors. The geometric simplicity of the phantom was meant to establish an upper limit on volume measurement accuracy. A threshold‐based segmentation algorithm was used to assure segmentation consistency, as well as to enable volume assessment over a wide array of different parameters.

## II. MATERIALS AND METHODS

### A. Phantom construction

A phantom was fabricated which contained 55 injection‐molded polymethylmethacrylate spheres (eleven different sizes with five‐fold repetition) embedded in gelatin, encased in an airtight rectangular polypropylene container. The container (Lock&Lock Co., Ltd., Seoul, Korea) measured 40.0 cm in height with a 15.2 cm by 15.2 cm square base. The spheres (McMaster‐Carr Supply Company, Los Angeles, CA) ranged in diameter from 1.6 mm to 25.4 mm. Prior to incorporation into the phantom, all 55 acrylic spheres were weighed using an electronic balance (Mettler AT261 Deltarange, Mettler‐Toledo, Inc., Columbus, OH) and each sphere's diameter was measured using digital calipers.

The spheres were incorporated into the phantom between approximately 6 cm thick layers of gelatin. Each layer of spheres included all eleven sphere sizes, resulting in five layers of spheres. The placement of spheres in each layer was different. Gelatin (Kraft Foods Global, Inc., Northfield, IL) was prepared to a concentration of 147.9 g/L in boiling water and poured over each layer of spheres. The phantom cooled for approximately 30 minutes at 4 °C after pouring each layer to allow the gelatin to harden. An example of a cross‐section through the phantom showing the spheres is presented in Fig. 1(A).

**Figure 1(A):**
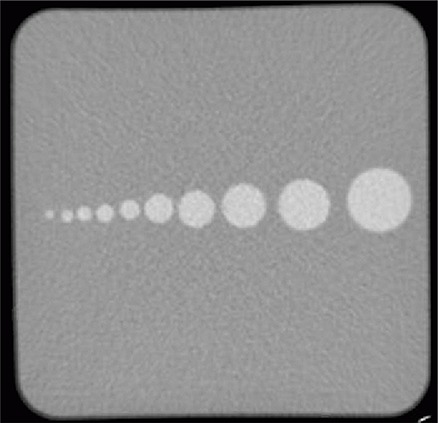
CT scan of phantom. 2D axial slice through phantom demonstrating PMMA spheres embedded in gelatin. Image taken from image set 1 (0.625 mm slice thickness).

### B. Phantom CT scanning

The phantom was scanned on a General Electric LightSpeed 16 multislice scanner (Waukesha, WI, U.S.A.). The phantom was positioned in the CT scanner once and all scan parameters were held constant at 120 kVp, 300 mAs, with 0.625 mm detector setting, 50 cm scan field of view and with a pitch of 0.938:1. The images were reconstructed using three different FOVs (20 cm, 30 cm, and 40 cm) and three different kernels (standard, lung, and bone). The scanning and reconstruction parameters for each image set studied are summarized in Table 1. The PMMA spheres and gelatin background had average Hounsfield units (HU) of 118.2 HU and 37.6 HU, respectively.

**Table 1:** Summary of scan and reconstruction parameters used to obtain image sets. In total, there were nine combinations of FOV and kernel, each evaluated with four different slice thicknesses, yielding 36 total image sets. The GE scanner used software version 07MW11.10_SP‐2‐26.H2_P_M16_G_ZEUS.

*Image Set*	*Reconstruction Parameters*			*Scan Parameters*		
	*FOV (cm)*	*Kernel*	*Slice Thickness (mm)*	*kVp*	*mA*	*Pitch*
1	20	Standard	0.625, 1.25, 2.5, and 5	120	300	0.938:1
2	20	Lung	0.625, 1.25, 2.5, and 5	120	300	0.938:1
3	20	Bone	0.625, 1.25, 2.5, and 5	120	300	0.938:1
4	30	Standard	0.625, 1.25, 2.5, and 5	120	300	0.938:1
5	30	Lung	0.625, 1.25, 2.5, and 5	120	300	0.938:1
6	30	Bone	0.625, 1.25, 2.5, and 5	120	300	0.938:1
7	40	Standard	0.625, 1.25, 2.5, and 5	120	300	0.938:1
8	40	Lung	0.625, 1.25, 2.5, and 5	120	300	0.938:1
9	40	Bone	0.625, 1.25, 2.5, and 5	120	300	0.938:1

#### C.1 Image processing

Segmentation of the implanted spheres from the phantom image sets was performed using a combination of the public domain Java‐based image processing program ImageJ version 1.40 (U.S. National Institutes of Health, Bethesda, MD) and segmentation code written in C++ (Visual Studio 2005, Microsoft Corporation, Redmond, Washington) at our institution. Segmentation of the smallest spheres (1.6 mm diameter) proved problematic, so they were eliminated in the subsequent analysis. The basic algorithm design included image preprocessing, segmentation of the spheres employing a global threshold, and volume measurements of each sphere. Through each combination of the three FOVs and three different kernels (standard, lung, or bone), nine image sets were produced. The native CT acquisitions were used to generate 0.625 mm thick images and CT images thicker than 0.625 mm were generated by averaging contiguous 0.625 mm images together. For example, two contiguous images were averaged to synthesize 1.25 mm thick images, four were used to generate 2.50 mm thick images, and so on. To assess the validity of averaging slices, we evaluated one clinical patient case scanned on a 16‐slice GE clinical scanner that simultaneously reconstructed thin‐slice (0.625 mm) images of the abdomen as well as thick‐slice (5.0 mm) images. Both reconstructions were derived from the same acquisition. The slice to slice separation was approximately 0.52 mm, effectively oversampling the object; so 9.625 slices were averaged to reproduce “virtual,” thick slices of 5.0 mm thickness. Linear interpolation was used for fractions of slices. We varied the phase of the slice averaging and identified the phase shift that minimized the magnitude of the subtraction of true thick‐slice images from the images created by averaging thin slices. In other words, the phase shift was simulated by averaging groups of 9.625 slices after skipping an initial set of between zero and nine slices. That initial set was averaged to yield the first “thick” slice.

#### C.2 Preprocessing

Initially, preprocessing of each image dataset was performed using the multiple image adjustment ImageJ commands. This included cropping out the air regions surrounding the scanned phantom in order to reduce processing workload and to allow better localization of segmentation. Windowing (400 HU) and leveling (0 HU) were then done to optimize viewing of these images. The resultant images were saved out in RAW format.

#### C.3 Segmentation

Segmentation was performed with code written in C++ using the open source library “NDL (The N‐Dimensional Library).” An initial analysis of the effects of grayscale threshold selection was performed on three cases. The image sets selected were the 0.625 mm slices of image set 1 (standard kernel, 20 cm FOV), 5 mm slices of image set 9 (bone kernel, 40 cm FOV), and 2.5 mm slices of image set 5 (lung kernel, 30 cm FOV), representing what was subjectively judged to be the smoothest case, the noisiest case, and an intermediate case, respectively. The analysis consisted of applying various threshold levels, segmenting the spheres, measuring their volume, and calculating the percent error in volume measurement for each sphere. The maximum, minimum, and median percent error calculated for the ten different sphere sizes were plotted as a function of threshold. The point at which the line of best fit for median percent error crossed the x‐axis (zero percent error) was recorded as the optimal threshold level. The optimal threshold levels for the three cases were averaged to yield a global optimal threshold level of 93 HU.

The optimal grayscale threshold found in the preliminary analysis was then applied to all 36 image data sets for the subsequent analysis. After thresholding, a 3D connected components algorithm was used to uniquely label all contiguous objects identified in the image dataset. The algorithm enforced a minimum volume threshold equal to ten percent of the smallest detected sphere volume (1.68 mm^3^), as well as a minimum connectedness threshold requiring each component voxel to have at least three neighbors to be included in an object. These requirements helped minimize the erroneous segmentation of artifacts as well as prevent multiple spheres from being identified as connected objects in noisy images. An example of an image (20 cm FOV, standard kernel, 0.625 mm slices) after segmentation is presented in Fig. 1(B).

**Figure 1(B):**
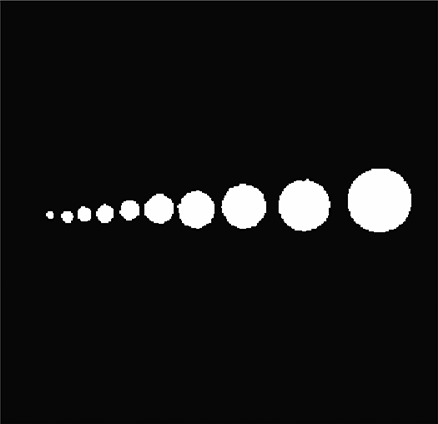
Segmented phantom. 2D axial slice through phantom after image processing and segmentation. Image taken from image set 1 (0.625 mm slice thickness).

### D. Volume measurement

The volume of each sphere was calculated by counting the number of voxels associated with a given object label and multiplying by the volume of a single voxel. The formula used to calculate each spherical volume $\left(V_{\text{CT}}\right)$ is shown in Eq. (1):

(1) $$V_{CT}=N_{V}\times l\times w\times t$$

where $N_{V}$ is the number of voxels associated with a given label, *l* is the individual voxel length, *w* is the individual voxel width, and *t* is the nominal slice thickness for the particular reconstruction image set. Ultimately, with three different FOVs, three different kernels, and four different slice thicknesses included in the study, a total of 36 image sets were analyzed. With 50 spheres measured in each image set, approximately 1800 volume measurements were performed by the segmentation algorithm.

### E. Statistical analysis

The sphere volumes measured using the segmentation algorithm were compared to the true volumes, as calculated from the diameter reported by the manufacturer, in order to obtain percent error. Percent error was calculated as:

(2) $$Percent\;Error=\frac{V_{CT}-V_{true}}{V_{true}}\times 100\%$$

where $V_{CT}$ was given by Eq. (1) and $V_{true}$ was calculated by the volume equation for a sphere, $\text{Vol}=(4/3)\pi r^{3}$. The volume error was calculated for all sphere sizes under the various combinations of CT imaging and reconstruction parameters. Precision in volume measurement was evaluated by calculating the 95% confidence interval and coefficient of variation (COV), defined as the standard deviation divided by the mean and represented as a percent, for the five replicates of each sphere size. Statistical significance for grouped data was performed using one way analysis of variance with Bonferroni correction for multiple comparisons. Descriptive statistics were calculated with standard spreadsheet software (Microsoft Excel, Microsoft Corporation, Redmond, Washington) and ANOVA was performed using STATA 11 (StataCorp LP, College Station, TX).

## III. RESULTS

### A. Preliminary analyses

The averaging of thin‐slice images to produce thick‐slice images was compared to true thick‐slice images in a clinical dataset. The “virtual” thick‐slice images created from averaging were subtracted from the true thick‐slice images. The average pixel intensity of the resulting subtraction image was approximately 0.3 HU. This corresponds to 0.03% of the maximum value (1000 HU).

A global grayscale segmentation threshold for the study was identified by performing a preliminary analysis on three image datasets representing the smoothest case (image set 1), the noisiest case (image set 9), and an intermediate case (image set 5). Each image set was thresholded at 90, 100 and, 110 HU. The median percent error for the complete set of sphere sizes was calculated and plotted as a function of threshold. At 90 HU, the median percent error was positive for image set 1 and image set 9. At 110 HU, the median percent error was negative for all three cases. The optimal grayscale threshold for each case was identified as the HU value, for which the line of best fit through the median percent error data points had zero error. Image set 1, image set 5, and image set 9 had optimal thresholds of 101, 78, and 101 HU, respectively. The optimal global threshold was calculated as the average of these three values (93 HU).

### B. Variability in volume error and general trends

Overall, the average error in volume measurement for any given sphere size ranged from −94.9% to −1.5%. The minimum error (−1.5%) was obtained for the 19.1 mm diameter sphere imaged with 0.625 mm slice thickness, lung kernel, and 20 cm FOV. The maximum negative error was obtained for the 6.4 mm diameter sphere imaged with 5.0 mm slice thickness, bone kernel, and 30 cm FOV. It must be noted that while all of the largest spheres were detected, many of the smaller sphere measurements were below threshold. For example, the 3.2 mm sphere was detected in 35% (63/180) of cases. The rate of detection increased with increasing sphere size and decreased with increasing slice thickness. The nondetection events were excluded during the analysis, although they did impact the calculated statistics, reflected by the standard deviation in percent error and 95% confidence intervals of volume measurement. As this is not a study of detection rate, the topic is not examined in greater detail, although perhaps could be in a future study.

When evaluating the median error for all of the sphere sizes imaged using any given combination of parameters, the median error varied from −43.2% to −4.9%. The maximum negative median error was obtained with 5.0 mm slice thickness, bone kernel, and 30 cm FOV. The minimum median error (−4.9%) was obtained with 0.625 mm slice thickness, lung kernel, and 20 cm FOV.

**Figure 2:**
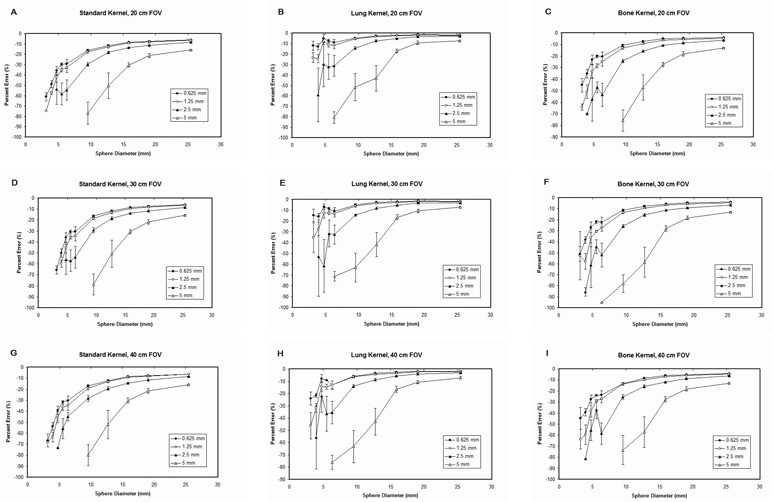
Percent error in sphere volume: estimated by CT with imaging parameters as defined by image set 1 (A); image set 2 (B); image set 3 (C); image set 4 (D); image set 5 (E); image set 6 (F); image set 7 (G); image set 8 (H); and image set 9 (I). Error bars represent standard error amongst the replicates of each sphere size.

In general, it was found that sphere size and percent error in volume were inversely proportional, with the error increasing as the sphere size decreased (Figs. 2(A) – 2(I)). The effect of sphere size on percent error was statistically significant (p<0.0001). As sphere diameter decreased, the standard deviation of percent error increased, yielding a larger spread and thus more variability in percent error.

**Figure 3:**
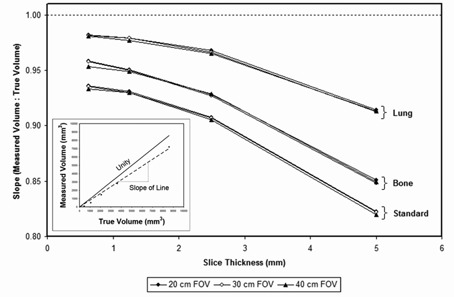
Accuracy as a function of slice thickness, kernel, and FOV. The average measured sphere volume was plotted against the true volume and the slope of the line of best fit was calculated (insert). The slope of this relationship was calculated for all combinations of parameters and plotted as a function of slice thickness. The lung kernel had the highest accuracy (ratio closest to one) and accuracy decreased with increasing slice thickness.

The true volume of the spheres was plotted against the measured sphere volume for each combination of imaging parameters. If the volume estimation was perfect, the plot of measured volume against the true volume would fall onto the line of unity. As such, the slope of the line of best fit through the data, forced though the origin, was used to describe accuracy (Fig. 3). Overall, for all combinations of imaging parameters, the slopes representing the ratio of measured volume to true volume ranged from 0.819 $\left(r^{2}=0.9999\right)$ for 5.0 mm slice thickness, standard kernel, and 40 cm FOV to 0.982 $\left(r^{2}=0.9999\right)$ for 0.625 mm slice thickness, lung kernel, and 20 cm FOV. The ratio of measured volume to true volume was less than unity for all parameter combinations.

### C. Effects of slice thickness on CT sphere volume measurement

The average percent error in volume measurement was calculated for all spheres measured under all combinations of slice thickness and other imaging parameters. It was found that error was largest when using large slice thickness and decreased as the slice thickness was narrowed. In Figs. 2(A)–2(I), percent error is plotted for each combination of imaging parameters with each curve representing a different slice thickness. All nine figures demonstrate worsening error with increasing slice thickness; error is progressively larger in magnitude and increasingly negative. The effect of slice thickness on percent error was statistically significant (p<0.0001).

The true volume of the spheres was plotted against the measured sphere volume for different slice thicknesses and the slope of the line of best fit was calculated. As shown in Fig. 3, as the slice thickness increased, the ratio of measured volume to the true volume (the slope of the line of best fit) decreased, deviating away from unity, for all combinations of kernel and FOV. To better observe the effects of slice thickness on small sphere (diameter≤6.4 mm) volume measurement, true volume was plotted against the measured volume in a log–log plot (not shown). In general the log–log plots mirrored the plots of percent error (Figs. 2(A)–2(I)), showing that the ratio of measured volume to true volume for small spheres dipped below the line of unity for the standard and bone kernel. The deviation from unity was progressively larger for larger slice thickness. For the lung kernel, the ratio of true volume to measured volume clustered close to unity for thin slices (0.625 mm and 1.25 mm), but deviated from unity for larger slice thickness.

### D. Effects of FOV on CT sphere volume measurement

Volume measurement accuracy was not significantly affected by FOV size (p=0.819). When comparing across all cases with standard kernel and varying FOV (Figs. 2(A), 2(D), and 2(G)), the minimum error, maximum error, and general trends with sphere size and slice thickness were all consistent. In fact, when collapsing the three plots into one, the effect of slice thickness dominates over any deviation between FOV. The same observations were made when comparing varying FOV for the lung kernel (Figs. 2(B), 2(E), and 2(H)) and for the bone kernel (Figs. 2(C), 2(F), and 2(I)). Similarly, Fig. 3 shows a clustering of curves based on kernel, but with very little variation within kernel type based on FOV. It must be noted that the differences in x‐y pixel dimension as a result of varying FOV were much smaller than the differences in slice thickness that were studied. The three FOVs (20 cm, 30 cm, and 40 cm) corresponded to square pixel dimensions of 0.391 mm, 0.590 mm, and 0.781 mm, respectively, whereas the slice thicknesses studied were 0.625 mm, 1.25 mm, 2.5 mm and 5 mm slices.

### E. Effects of kernel on CT sphere volume measurement

Kernel type had a strong effect on the pattern of volume measurement error (p<0.0001). All three kernels showed an increasingly negative percent error with decreasing sphere diameter. However, the lung kernel was least affected by decreasing sphere size, particularly for thin slices. In general, the lung kernel produced a noisier error profile as a function of sphere diameter, with larger standard error bars, than the standard and bone kernels (Figs. 2(A))–2(I)).

The slope of the line defined by the ratio of measured volume to true volume was plotted as a function of slice thickness for the three kernel types (Fig. 3). The slope of measured to true volume as a function of slice thickness clustered according to kernel. The lung kernel consistently had the largest slope, followed by the bone kernel, and then the standard kernel. For thin slices, the lung kernel was closest to unity.

### F. Volume measurement precision

Volume measurement precision was quantified by calculating the 95% confidence intervals and COV for the five replicate volume measurements at each sphere size. For all sphere sizes, the range of the 95% confidence interval increased with slice thickness. Similarly, the COV increased as slice thickness increased and decreased with larger sphere diameters. For the specific case of a 30 cm FOV, the bone kernel and 1.25 mm slices, the 95% confidence intervals for measured volume were 0.8–15.1 mm^3^ for the 3.2 mm diameter sphere $\left(\text{true volume}=16.8\;{\text{mm}}^{3}\right)$, 89–107 mm^3^ for the 6.4 mm sphere $\left(\text{true volume}=134\;{\text{mm}}^{3}\right)$, and 8131–8236 mm^3^ for the 25.4 mm diameter sphere $\left(\text{true volume}=8580\;{\text{mm}}^{3}\right)$. The respective COVs were 65%, 10%, and 0.7%. All spheres of diameter greater than or equal to 9.5 mm in this case had a COV less than 5%. Volume measurement precision for three representative sphere sizes is presented in Table 2.

**Table 2:** Volume measurement precision was measured using the coefficient of variation of the five replicate measurements of each sphere size at a given slice thickness, FOV, and kernel. The precision for three representative sphere diameters (4.8 mm, 9.5 mm, and 19.1 mm) is presented. Spheres that were not detected under a given set of scan parameters were assigned a COV of N/A.

*FOV*	*Kernel*	*4.8 mm Sphere Slice Thickness (mm)*				*9.5 mm Sphere Slice Thickness (mm)*				*19.1 mm Sphere Slice Thickness (mm)*			
		*0.625*	*1.25*	*2.5*	*5*	*0.625*	*1.25*	*2.5*	*5*	*0.625*	*1.25*	*2.5*	*5*
**20 cm**	**Standard**	15.1	18.3	44.4	N/A	2.9	3.2	6.3	81.1	1.9	2.0	1.9	6.1
	**Bone**	12.2	20.4	62.9	N/A	2.5	2.7	4.6	76.9	1.4	1.4	1.6	5.5
	**Lung**	9.5	7.2	53.1	N/A	1.9	1.8	3.3	53.7	1.1	1.1	1.2	4.4
**30 cm**	**Standard**	14.5	18.8	43.9	N/A	3.0	3.4	7.3	77.3	1.9	2.0	1.9	6.1
	**Bone**	15.2	29.5	74.9	N/A	2.7	3.4	4.3	83.6	1.5	1.6	1.5	4.4
	**Lung**	6.3	11.9	110.4	N/A	2.1	2.3	1.8	80.1	1.1	1.0	1.4	4.1
**40 cm**	**Standard**	13.7	21.3	N/A	N/A	2.7	4.2	8.3	82.9	1.8	2.0	2.4	6.1
	**Bone**	11.1	19.7	30.0	N/A	2.0	3.2	6.4	86.1	1.5	1.5	1.8	5.6
	**Lung**	8.1	10.1	24.4	N/A	2.4	1.7	3.3	80.2	1.1	1.0	1.2	3.5

## IV. CONCLUSIONS

The effects of various CT parameters such as window setting, slice thickness, segmentation threshold, field of view, peak voltage, and tube current on volumetric accuracy have been previously documented.^(^ ^13^ ^–^ ^16^ ^)^ As predicted, optimal adjustment of parameters that affect spatial resolution – most prominently slice thickness – improves volumetric accuracy.^(17)^ In this study, the accuracy and precision of spherical object volume assessment was determined under near optimal conditions of a small diameter homogeneous object and high dose acquisition. While the segmentation technique used in this study was based on a simple grayscale threshold, an optimal threshold was selected to generalize the method to all cases. Thin‐slice images were averaged to produce thick‐slice images. The preliminary analysis of this technique showed an average pixel intensity difference between true thick‐slice acquisition and averaged thin CT slices of approximately 0.3 HU, or 0.03% of the maximum value (1000 HU). Using the preliminary evaluation of our thresholding technique, described in the methods section, a change in grayscale threshold of 0.3 HU corresponds to a change in median percent error by 0.4%. This is less than the minimum volume reproducibility reported (0.7%). While averaging thin slices may not be equivalent to thick‐slice CT acquisition, the 0.03% difference in grayscale HU affects the percent error in volume measurement less than the reproducibility of volume measurement itself. The simplicity of the phantom design used in this study establishes an upper limit in volume measurement accuracy. A lower degree of accuracy would be expected in the actual clinical setting, where tumors are nonspherical and the patient images contain more complex anatomical background. Nevertheless, the results obtained here serve as reasonable guidance for the tumor volume accuracy that could be expected under optimized clinical protocols.

The analysis of percent error (Figs. 2(A)–2(I)) shows that CT enjoys greater volumetric accuracy for larger spheres that are imaged with thinner slices. Given the large average percent error for the 2.5 mm and 5.0 mm slice thicknesses, it is apparent that thin CT slices (0.625 mm or 1.25 mm) should be used when volume assessment is anticipated in clinical CT scanning. For thin‐slice scenarios, accuracy of better than 20% can be achieved for object diameters on the order of 10 mm and larger; however, it is clear that volumetric accuracy degrades for spherical lesions smaller than 10 mm in diameter. These values are improved, achieving 20% error with objects as small as 5 mm in diameter, when the lung kernel is used instead of the standard or bone kernel.

The results demonstrate that, with the given imaging and segmentation parameters, the measured volume of smaller spherical objects tends to be underestimated relative to the actual volume. Figures 2(A)–2(I) illustrate the progressive underestimation of volume for small sphere sizes. The effect is amplified by the use of large slice thickness. The slope of the line representing the ratio of measured sphere volume to true sphere volume also reflected an overall underestimation of volume. Figure 3 shows the measured slope as less than one (underestimating volume) for all of the combinations of parameters. This underestimation worsened with increasing slice thickness. The degree of underestimation is strongly dependent on threshold selection. Although an optimal threshold was selected for the methods in this study, threshold selection for other unique scenarios should be studied in the future.

Precision measurements were also obtained. As in the case of volume accuracy, the precision of volume measurement improved for larger sphere sizes imaged with thinner slices. When using thin slices (0.625 or 1.25 mm), an approximately 10 mm sphere diameter may serve as a cutoff between high precision with a COV less than 5% versus an exponentially increasing COV and drop in precision for spheres below that threshold. Depending on the acceptable level of precision, this threshold may be lowered to 5.7 mm spheres (COV=15%) or 4.9 mm spheres (COV=20%).

Similarly, the results from this study provide guidance in predicting the minimum sphere size whose volume can be measured within a specific error tolerance, given a set of scan parameters. Such a summary is presented in Table 3. As a clinical scenario, one can imagine a situation in which a patient may receive a CT scan as part of the workup for a solitary pulmonary nodule. Given the patient's body size, a minimum FOV would be selected (e.g. 30 cm). The CT image protocol might call for thin slices (0.625 mm) and processing with the lung kernel. Table 3 suggests that any detected lesion with an equivalent spherical volume of diameter greater than 6.9 mm would have a measured volume with no more than 10% error from the true lesion volume. Furthermore, the results of this study and the summary in Table 3 may have radiation dose implications. Given a maximum error tolerance, Table 3 allows one to identify situations in which larger slice thickness can be used, sparing dose, without what may be deemed a clinically relevant loss of accuracy.

**Table 3:** Lookup table of imaging parameters, and the minimum sphere diameter (in mm) that has no more than 10% and 20% expected volume measurement error.

*FOV*	*Kernel*	*10% Volume Error Slice Thickness (mm)*				*20% Volume Error Slice Thickness (mm)*			
		*0.625*	*1.25*	*2.5*	*5*	*0.625*	*1.25*	*2.5*	*5*
**20 cm**	**Standard**	14.2	15.2	21.9	>25.4	8.6	9.1	12.2	20.4
	**Bone**	10.2	12.1	17.1	>25.4	6.5	7.6	11.1	18.4
	**Lung**	4.3	7.2	11.5	18.7	<3.2	4.2	8.5	15.5
**30 cm**	**Standard**	14.4	15.4	22.2	>25.4	8.7	9.2	12.3	20.8
	**Bone**	10.6	12.3	17.7	>25.4	7.0	8.0	11.3	18.5
	**Lung**	6.9	7.7	11.7	20.0	<3.2	4.4	8.5	15.4
**40 cm**	**Standard**	14.6	15.3	22.4	>25.4	8.8	9.3	12.5	20.9
	**Bone**	11.7	12.6	17.9	>25.4	7.5	8.0	11.3	18.4
	**Lung**	7.7	7.8	11.9	20.4	4.1	4.5	8.6	15.5

The results from this study confirm the well‐established trends regarding the effects of slice thickness and object size on volume measurement accuracy. We also show that FOV does not have a significant effect on volume accuracy, as previously reported.^(17)^ The results comparing reconstruction kernels suggest that the lung kernel is the most accurate kernel, followed by the bone kernel and then the standard kernel. The lung kernel was found to have the noisiest error profile. While the phantom design and segmentation technique used in this study were simple and idealized, the results provide an upper limit of optimal expected accuracy. Future studies must evaluate more clinically‐applicable automatic and semiautomatic segmentation techniques and their effects on volume measurement. Our results still serve as an initial guide to estimate volumetric accuracy of clinical volume measurements, and also to aid in balancing volume accuracy considerations with dose optimization in clinical CT scan protocol design.

## ACKNOWLEDGEMENTS

Thank you to Nathan Packard for providing his open source C++ library “NDL (The N‐Dimensional Library)” and Dennis Belisle for assistance in scanning the phantom. This work was funded in part by a grant from the National Institutes of Health (R01 EB002138) and by a grant from the National Center for Research Resources (UL1 RR024146).
